# Impact of PSMA-Based Radiopharmaceuticals on the Clinical Management of Prostate Cancer

**DOI:** 10.3390/cancers18111799

**Published:** 2026-06-01

**Authors:** Cesare Guida, Laura Evangelista, Marco Spadafora, Gaetano Facchini, Luigi Mansi

**Affiliations:** 1 Radiotherapy Complex Unit, Ospedale del Mare, Azienda Sanitaria Locale Napoli 1 Centro, 80147 Napoli, Italy; cesare.guida@aslnapoli1centro.it; 2 Department of Biomedical Sciences, Humanitas University, Via Rita Levi Montalcini 4, Pieve Emanuele, 20072 Milan, Italy; 3 Nuclear Medicine Unit, IRCCS Humanitas Research Hospital, Via Manzoni 56, Rozzano, 20089 Milan, Italy; 4 Medicina Futura, 80143 Napoli, Italy; marco.spadafora@medicinafutura.it; 5 Oncology Complex Unit, Santa Maria delle Grazie Hospital, Azienda Sanitaria Locale Napoli 2 Nord, 80078 Pozzuoli, Italy; gaetano.facchini@aslnapoli2nord.it; 6 Interuniversity Research Center for Sustainability (CIRPS), 00038 Rome, Italy; mansi.luigi@libero.it

**Keywords:** prostate cancer, PSMA, PET/CT, radiotherapy

## Abstract

Background: Prostate cancer (PCa) is biologically heterogeneous, requiring management strategies that balance oncologic benefit with preservation of quality of life. Prostate-specific membrane antigen (PSMA) has emerged as a key theranostic biomarker enabling highly sensitive molecular imaging and targeted therapy. Purpose: The present manuscript aims to summarize the clinical role of PSMA-PET/CT in PCa across staging, treatment selection, and response assessment, with a special focus on its contribution to personalized management. Key Findings: PSMA-PET/CT demonstrates superior accuracy compared with conventional imaging, frequently leading to stage migration and changes in therapeutic strategy. It improves detection of metastatic and recurrent disease, guides selection for systemic and PSMA-targeted therapies, and supports metastasis-directed treatment in oligometastatic settings, potentially delaying androgen-deprivation therapy and preserving quality of life. Additionally, PSMA-PET enhances intra-prostatic lesion delineation for focal therapies and radiotherapy planning. Limitations include reduced sensitivity for very small lesions, possible false positives, variability among tracers, and issues related to access and standardization. Conclusions: PSMA-PET/CT is a cornerstone of precision imaging in PCa, enabling more individualized treatment decisions across the disease continuum. Ongoing studies will further define its long-term clinical impact and integration into routine care.

## Simple Summary

Prostate cancer can be managed using a range of therapeutic strategies, from the local (such as radiation therapy or focal treatments) to systemic ones. The introduction of next-generation imaging, mainly PSMA-based PET/CT has significantly changed the diagnostic workflow, as well as the selection of targeted therapies, including both systemic and localized treatments. In this context, we discuss the use of PSMA primarily for guiding radiation therapy in case of oligometastatic disease, in which it has demonstrated meaningful benefits for patients’ quality of life. Moreover, a large overview was made regarding the results from the clinical trial, specifically in guiding focal treatments that represent an important alternative in case of localized and indolent disease. Finally, a large overview was made about the future of PSMA PET in prostate cancer patients, with an important emphasis on the standardization of interpretation and cures.

## 1. Background

Cancer is a very complex disease and the second leading cause of death globally. Several factors have been associated with the growth and progression of different types of cancer, including chemical, physical, and biological carcinogens. Numerous diagnostic and conventional methods are available for detection, management and treatment [1,2,3]. Prostate cancer (PCa) is the second most detected cancer in males globally, with 396,792 deaths and 1,466,680 new cases in 2022. Several factors are involved in the progression of PCa, such as the environment and genetic alterations [4,5,6].

The growing emphasis on personalized medicine highlights the need to tailor PCa management to individual patient priorities, including the preservation of quality of life (QoL), even when doing so may modestly compromise life expectancy. PCa exhibits substantial biological heterogeneity: while curative strategies are often effective, they may also produce significant morbidity. Conversely, many prostate tumors follow an indolent course, raising concerns regarding unnecessary treatment. The delineation between clinically significant and indolent disease therefore remains central to patient management, particularly in the context of demographic aging.

Prostate-specific membrane antigen (PSMA) has emerged as an important theranostic biomarker, offering high specificity for prostate tissue and enabling both precise molecular imaging and targeted radionuclide therapy [7,8]. PSMA-based approaches enhance risk stratification and support individualized decisions across the entire PCa continuum, from diagnosis and staging through surveillance, treatment selection, and response assessment [9].

In the following paragraphs, we aim to describe the utility of PSMA PET/CT in patients with PCa by analyzing selected clinical settings that represent the future development of next-generation imaging in this highly prevalent male disease. The discussion is intentionally narrative rather than systematic, reflecting insights derived from clinical practice and our daily experience.

## 2. Epidemiology, Screening, and Surveillance

PCa is the second most diagnosed malignancy among men globally [10]. Despite widespread use of PSA testing, population-based screening remains controversial. Although screening increases cancer detection rates, demonstrated benefits in overall or cancer-specific survival remain inconclusive. However, overdiagnosis and overtreatment represent key concerns, underscoring the importance of personalized management pathways. Management strategies for low- and intermediate-risk disease include (i) watchful waiting, a symptom-driven conservative approach recommended for patients with limited life expectancy or those unsuitable for curative therapy [11]; (ii) active surveillance, a structured monitoring protocol for individuals with low-risk localized disease and life expectancy ≥10 years, combining PSA testing, imaging, and repeat biopsies [12]; and (iii) active monitoring, a less intensive PSA-based follow-up strategy used in selected patients [13]. These three approaches have a common endpoint: to avoid treatments in patients who cannot benefit from them while minimizing adverse effects on QoL.

Indeed, definitive local therapies, such as radical prostatectomy and external-beam radiotherapy, may produce both early and late toxicity affecting urinary, sexual, and bowel function [14]. Systemic therapy with androgen-deprivation therapy (ADT) is associated with fatigue, sexual dysfunction, psychological distress, metabolic syndrome, bone demineralization, and increased cardiovascular risk [15]. These adverse effects must be carefully balanced against therapeutic benefit, particularly in older or frail patients. Therefore, a personalized approach to PCa management is urgently needed, particularly in the context of an aging population.

## 3. Mechanism of PSMA

**Figure 1:**
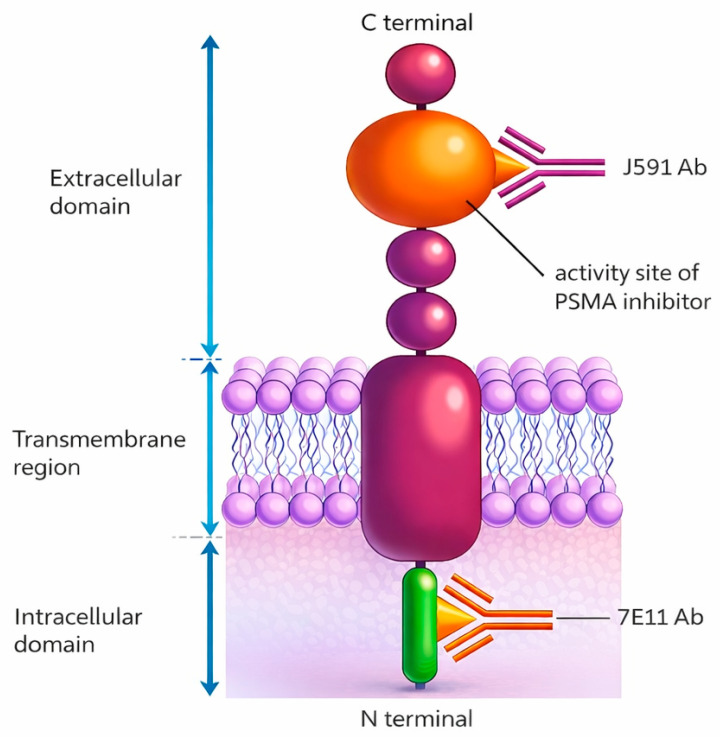
A schematic illustration of PSMA mechanisms.

PSMA is a type II transmembrane glycoprotein highly overexpressed on prostate cancer cells, particularly in advanced, metastatic, and castration-resistant disease. Its extracellular domain functions as a glutamate carboxypeptidase (also known as folate hydrolase), enabling ligand binding with high specificity. Upon binding of small-molecule inhibitors or radiolabeled ligands, the PSMA–ligand complex undergoes clathrin-mediated internalization, resulting in intracellular accumulation [16]. While PSMA is physiologically expressed in normal prostate epithelium, salivary glands, proximal renal tubules, and small intestine, its expression is significantly upregulated in PCa, with reported increases of up to 100–1000-fold compared to benign tissue. This property forms the biological basis for both diagnostic imaging (i.e., PSMA-PET) and targeted radionuclide therapy, as radioactive payloads are selectively delivered and retained within tumor cells while sparing most normal tissues. Additionally, PSMA expression is upregulated by androgen deprivation, further enhancing target availability in advanced disease [17]. Figure 1 presents a schematic representation of how PSMA targets PCa cells. PSMA, also known as glutamate carboxypeptidase II (GCPII) or folate hydrolase 1 (FOLH1), is a type II transmembrane glycoprotein composed of a short intracellular domain, a transmembrane segment, and a large extracellular catalytic domain.

## 4. Role of PSMA PET/CT in Clinical Management

**Figure 2:**
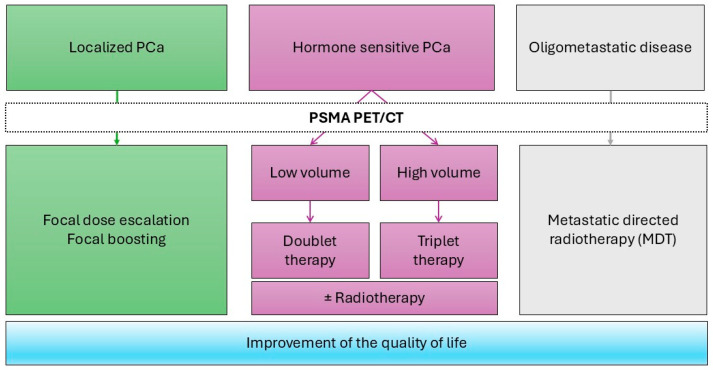
A schematic summary of the use of PSMA PET in diverse settings.

PET/CT with [68Ga]Ga- or [18F]F-labeled PSMA ligands has rapidly become a powerful tool for staging and restaging due to its high sensitivity and favorable biodistribution. Validated indications include (1) initial staging of unfavorable intermediate- to high-risk PCa; (2) localization of biochemical recurrence or persistence after curative therapy; (3) evaluation of non-metastatic castration-resistant PCa following negative conventional imaging and (4) pre-treatment assessment for PSMA-targeted radioligand therapy [7,8]. Emerging studies suggest potential roles within active surveillance protocols and in guiding initial intra-prostatic lesion characterization, although broader implementation awaits further validation [9]. In Figure 2, the settings of disease and PSMA utility are reported. The image illustrates the role of PSMA PET/CT in guiding management across three major clinical settings of PCa: localized disease, hormone-sensitive disease, and oligometastatic disease. In patients with localized PCa, PSMA PET/CT improves intraprostatic tumor localization, enabling more precise treatment planning. In this context, it supports strategies such as focal dose escalation or focal boosting, with the aim of improving local control while minimizing toxicity. In the setting of hormone-sensitive PCa, PSMA PET/CT allows for more accurate disease burden stratification, distinguishing between low-volume and high-volume metastatic disease. This distinction has direct therapeutic implications: patients with low-volume disease are typically candidates for doublet systemic therapy, whereas those with high-volume disease may benefit from intensified triplet regimens. In both cases, PSMA imaging may also inform the addition of radiotherapy to the primary tumor or metastatic sites. For patients with oligometastatic disease, PSMA PET/CT enables sensitive detection of a limited number of metastatic lesions, thereby supporting the use of metastasis-directed therapies (MDT), such as stereotactic body radiotherapy (SBRT), with the goal of delaying systemic treatment escalation. Overall, the integration of PSMA PET/CT into these clinical scenarios facilitates more personalized treatment strategies, ultimately contributing to an improvement in patients’ QoL.

Although promising, PSMA-PET/CT has a limited detection rate for PSA values below 0.2 ng/mL (about 30% for any radiolabeled peptides, with or without physiological bladder uptake) [18]. Indeed, in this context, in case of radical prostatectomy, salvage radiotherapy should be performed independently from the PSMA-PET positivity [19]. A major role could be played by MRI in this setting of disease, although limited data are available for low PSA values. In this context, clinical trials are urgently needed. Unknown bone uptakes (UBUs) are often reported for all types of PSMA agents, mainly for [18F]F-PSMA-1007, thus negatively altering the detection rate, with the risk of overtreatment/overdiagnosis [20]. In order to overcome these confounding results, CT coregistered images and MRI scan would be useful for the correct differentiation between benign and malignant findings. Also, clinical data would always be taken into consideration.

## 5. PSMA PET/CT to Guide Treatment Decision-Making

### 5.1. Influence on Systemic Therapy Selection

PSMA-PET/CT has substantially improved the detection of metastases at initial diagnosis, resulting in notable stage migration [21]. This has implications for the classification of hormone-sensitive metastatic PCa (mHSPC), which traditionally relies on CHAARTED and LATITUDE criteria derived from conventional imaging. Because PSMA-PET identifies smaller deposits, many patients are upstaged to metastatic disease, potentially altering the appropriateness of doublet or triplet systemic regimens or primary-tumor radiotherapy. Conversely, its specificity can significantly affect the overdiagnosis provided by conventional imaging techniques, such as bone scans [22]. A multicenter series demonstrated that 22% of patients classified as low-volume by conventional imaging were reclassified as high-volume by PSMA-PET, while nearly a third were downstaged to non-metastatic disease—underscoring the modality’s reclassification power [21,22]. The identification of stage migration with PSMA-PET, as demonstrated by the ProPSMA trial [23], and the effect, provided by the VISION trial [18], for the selection of patients to be submitted to radioligand therapy based on PSMA are two great advantages obtained by receptorial imaging. We analyzed preliminary data regarding the utility of PSMA-PET/CT as baseline imaging for monitoring the response to therapy with advanced mHSPCa. Indeed, in 40 patients, we found that total PSMA-positive lesion burden was significantly correlated with PSA values, and the best response to PSA was correlated with a significant reduction in total PSMA-positive lesions. Furthermore, progressive disease was more often associated with a large total number of PSMA-positive lesions. The data were discussed in the annual congress of the European Association of Nuclear Medicine in 2024 [24]. The collection of data is still ongoing to include a large patient population, and results will be published in the upcoming months.

Patient selection for a precise treatment strategy can be considered useful in case of alternative approaches. To date, PSMA-PET plays an important role in the identification of receptor expression, its heterogeneity, and its tumor volume and for monitoring the efficacy of therapies [25]. In clinical practice, PSMA-PET/CT is widely used to evaluate the expression of receptors to choose the best candidates for PSMA-based radioligand therapy; however, large variability in terms of criteria has also been reported across the diverse clinical trials. In Table 1, some studies are reported relative to the utility of PSMA in guiding systemic treatments. This table summarizes major trials demonstrating the impact of PSMA PET on clinical decision-making in PCa. PSMA PET improves staging accuracy (proPSMA), enables selection of patients for targeted radioligand therapy (VISION, TheraP), and significantly influences management in recurrent disease (CONDOR, EMPIRE-1). Across settings, its use frequently leads to earlier initiation, intensification, or modification of systemic therapy, supporting more personalized treatment strategies.

### 5.2. Oligometastatic Disease and MDT

The oligometastatic state (commonly defined as ≤5 lesions) represents a window in which MDT may delay systemic treatment. The introduction of next-generation imaging has significantly increased the detection of OMD or oligorecurrent disease, thus prolonging the time free of systemic treatment.

PSMA-PET excels at identifying limited metastatic disease, enhancing patient selection for MDT [30]. Randomized evidence from the ORIOLE trial confirms that MDT guided by PSMA-PET prolongs progression-free survival [31]. Additional trials (LIGHTHOUSE, OSPREY [32,33] show that PSMA-PET reveals metastatic disease missed by conventional imaging, facilitating earlier intervention. The EAU recognizes MDT as a strategy to defer ADT initiation, improving QoL. Indeed, this practice can be useful both in patients at the hormone-sensitive stage and in those in castrate-resistant conditions, thus improving both local control and potential symptoms.

Table 2 lists the list of clinical trials related to the use of PSMA-PET for the detection of OMD, which is able to guide local therapy and to delay systemic treatment. There are some key studies supporting the role of PSMA PET in guiding MDT. PSMA PET improves detection of occult disease and refines patient selection, as shown in ORIOLE and Oligo-PELVIS. It enhances staging accuracy (OSPREY) and enables precise targeting of nodal and metastatic lesions, leading to improved outcomes. Ongoing trials such as LIGHTHOUSE are further evaluating its role as a companion diagnostic to guide early integration of PSMA-targeted systemic therapies.

### 5.3. Intra-Prostatic Dose Escalation

The prostate gland is anatomically divided into distinct zones such as the peripheral zone (PZ), the transition zone (TZ), and the central zone (CZ), each characterized by different proportions of glandular and stromal components. The PZ, which constitutes approximately 70–75% of the glandular tissue in young men, is predominantly composed of glandular elements with relatively sparse stroma. In contrast, the TZ (5–10% in young individuals, increasing with age due to benign prostatic hyperplasia) has a more balanced glandular-to-stromal ratio, while the CZ (approximately 20–25%) contains a higher proportion of stromal tissue surrounding the ejaculatory ducts. Notably, about 70–80% of prostate cancers arise in the peripheral zone, likely due to its higher density of secretory epithelial cells and greater exposure to carcinogenic and inflammatory stimuli. This zonal predilection underpins the rationale for imaging-guided, focal therapeutic approaches, aimed at selectively targeting dominant intraprostatic lesions (DILs) while sparing surrounding normal tissue.

Focal dose escalation to DILs improves biochemical control, as demonstrated in the FLAME trial [35]. However, MRI alone is limited by inter-reader variability and potential for lesion under-detection. PSMA-PET provides complementary biologic characterization and often delineates larger gross tumor volumes. Trials such as HypoFocal and ARGOS/CLIMBER are evaluating integrated PSMA-PET/MRI guidance for focal boosting [36]. Early findings suggest improved tumor delineation and potential enhancements in local control. However, the limited available data renders this approach experimental.

PSMA-PET/CT has an important role in guiding focal therapies, not only in radiation treatment approaches, but also in High-Intensity Focused Ultrasound (HIFU). Some preliminary data about this latter approach have been recently discussed in the last European Association of Nuclear Medicine (EANM) congress [37]. From our own institutional experience, we found that after 12 months of focal procedures, patients with a primary score equal to 4 and 5 (*n* = 37/48 enrolled subjects) at PSMA-PET/CT showed a reduction in PSA to 58% and 75%, respectively. Additional analysis is still ongoing for establishing the utility of PSMA for guiding and monitoring the response to focal therapies in a larger population, and the results will be published in the upcoming months.

A list of ongoing or published trials has been reported in Table 3. The table summarizes studies exploring the role of PSMA PET in guiding focal and MDT. PSMA PET improves lesion detection and delineation, enabling more accurate targeting in both oligometastatic (BULLSEYE) and localized disease (PROBE, PRIMARY). Early clinical experiences, such as the UCLA cohorts, further support its value in enhancing the precision of focal therapies compared to conventional imaging. Ongoing trials will clarify its impact on treatment outcomes and its role as a standard tool for treatment planning.

## 6. Future Directions

Despite its substantial clinical utility, PSMA PET/CT presents several limitations that should be carefully considered in clinical practice. Sensitivity decreases for lesions smaller than 5 mm, and false-positive uptake may occur in benign or inflammatory conditions. Diagnostic performance may also vary among tracers; for example, [^18^F]F-PSMA-1007 has been associated with increased non-specific bone uptake [20]. In addition, access to PSMA imaging remains uneven across regions, and its cost-effectiveness is still under evaluation [42]. Variability in acquisition protocols and interpretation criteria further contributes to interobserver variability, underscoring the need for standardized imaging frameworks. In this context, structured reporting systems have been developed to improve reproducibility and clinical communication. PSMA-RADS provides a five-point scale analogous to BI-RADS or PI-RADS, facilitating standardized interpretation and transparent reporting of uncertainty [43]. Similarly, the PROMISE framework introduces molecular TNM classification (miTNM), integrating lesion location, extent, and PSMA expression relative to reference organs [44]. Despite their complementary roles, neither system has yet achieved universal adoption, and interpretative variability persists, particularly in low-uptake lesions, treated sites, and atypical metastatic patterns. Ongoing harmonization efforts are therefore essential, not only for clinical consistency but also for regulatory approval and reproducibility in clinical trials [45]. In this regard, the recent update from the Prostate Cancer Working Group 4 (PCWG4) represents an important step toward the broader integration of PSMA PET into standardized clinical pathways.

Beyond technical considerations, the expanding use of PSMA PET/CT across multiple clinical scenarios—including surveillance, focal therapy planning, and treatment response assessment—raises concerns regarding potential overutilization and increasing healthcare costs. Comprehensive health-economic evaluations incorporating long-term outcomes and quality-adjusted life years (QALYs) are still evolving and will be critical to define its sustainable implementation.

Access to PSMA PET/CT remains highly heterogeneous worldwide, reflecting disparities in infrastructure, regulatory approval, radiochemistry capabilities, and reimbursement policies [46,47,48]. While high-income countries are progressively incorporating PSMA imaging into clinical guidelines, access may still be limited in rural areas due to centralized production facilities. In contrast, middle- and low-income settings often face substantial logistical and economic barriers, including limited cyclotron availability and restricted distribution networks, further widening disparities in precision oncology care.

Ongoing prospective trials, such as PATRON and THUNDER, are expected to better define the long-term clinical impact and optimal indications of PSMA PET/CT [49,50]. In parallel, emerging innovations—including biology-guided radiotherapy and quantitative PSMA PET biomarkers—may further enhance prognostic stratification and support more refined treatment personalization. As highlighted in Table 1, Table 2 and Table 3, these developments will be instrumental in consolidating the role of PSMA PET within future clinical practice.

## 7. Conclusions

PSMA PET/CT has emerged as a central component in the management of PCa, offering superior diagnostic performance and enabling increasingly personalized therapeutic strategies. Its clinical utility spans the entire disease continuum, from initial staging and early detection of recurrence to treatment selection, metastasis-directed approaches, and precision radiotherapy planning, including focal dose escalation. Beyond its diagnostic role, PSMA PET/CT is progressively reshaping clinical decision-making by refining risk stratification and guiding therapy selection according to disease extent and biological characteristics. However, challenges related to accessibility, standardization, and cost, as well as the need for prospective outcome-driven evidence, remain important considerations.

Ongoing clinical trials and advances in imaging standardization and quantitative biomarkers are expected to further consolidate its role within routine practice. As these data mature, PSMA PET/CT is likely to become fully integrated into precision oncology frameworks, supporting optimized treatment pathways and outcomes that balance oncologic control with preservation of QoL.

## Figures and Tables

**Table 1:** List of trials regarding PSMA as a predictive biomarker in systemic treatment.

Trial, Ref	Year	Population	Role of PSMA PET	Key Finding	Impact on Systemic Therapy
proPSMA, [23]	2020	High-risk localized PCa (pre-treatment staging)	Compared PSMA PET vs. CT + bone scan	Accuracy 92% vs. 65%; management changed in 28%	Upstaging → earlier initiation or escalation of systemic therapy
VISION, [26]	2021	mCRPC after ARPI + taxane	PSMA PET used to select patients for Lu-177-PSMA	Improved OS and PFS in PSMA-positive patients	Determines eligibility for PSMA-targeted systemic therapy
TheraP, [27]	2021	mCRPC	PSMA PET used as gatekeeper for therapy	Lu-PSMA superior to cabazitaxel in selected patients	Imaging directs choice between chemo vs. targeted systemic therapy
CONDOR, [28]	2021	Biochemical recurrence	Assessed impact on management decisions	Management changed in 64% of patients	Frequently altered decision to start, intensify, or change systemic therapy
EMPIRE-1, [29]	2021	Biochemical recurrence post-RT	Molecular imaging guided treatment planning	Improved progression-free survival	Imaging-guided intensification of systemic and combined therapies

mCRPC = metastatic castrate resistant prostate cancer; ARPI = androgen receptor pathway inhibitors; RT = radiation therapy; OS = overall survival.

**Table 2:** List of trials related to the detection of OMD at PSMA PET/CT.

Trial, Ref	Year	Population	Imaging	Design	Intervention	Key Results	Clinical Implication
ORIOLE, [31]	2020	Recurrent hormone-sensitive PCa with ≤3 mets	Conventional imaging + PSMA-PET (post hoc)	Randomized (MDT vs. observation)	SABR to all detected mets	Patients with untreated PSMA-avid lesions had significantly worse PFS	PSMA-PET improves patient selection and completeness of MDT
Oligo-PELVIS GETUG P07, [34]	2021	Pelvic nodal oligorecurrence	Mostly PSMA-PET	Phase II	Whole pelvis RT + MDT + short ADT	2-yr PFS ~77%	PSMA-PET enables accurate nodal targeting
OSPREY, [33]	2021	High-risk localized PCa (cohort A) and metastatic PCa (cohort B)	Diagnostic validation using ^18^F-DCFPyL PSMA-PET	Phase II/III	Accuracy of PSMA-PET for nodal and distant staging	High specificity for nodal disease (>95%); improved detection vs. conventional imaging	Improves staging → impacts choice between local therapy, MDT, and systemic therapy
LIGHTHOUSE, [32]	Ongoing (2022–)	Metastatic hormone-sensitive PCa (mHSPC)	PSMA-PET used for patient selection (PSMA expression required)	Phase II	Early use of PSMA radioligand therapy (^225^Ac-PSMA-I&T) + abiraterone vs. abiraterone alone	Evaluates whether PSMA-targeted therapy improves outcomes when introduced earlier	Establishes PSMA-PET as a companion diagnostic guiding access to intensified systemic therapy

MDT = metastatic direct therapy; SABR = stereotactic ablative body radiotherapy; RT = radiation therapy; ADT = androgen-deprivation therapy; PFS = progression-free survival.

**Table 3:** Ongoing clinical trials regarding oligometastatic disease.

Trial/Study	Year	Modality	Population	Role of PSMA PET	Intervention	Key Findings	Clinical Relevance
BULLSEYE, [38]	Ongoing	MDT/focal RT	Oligometastatic recurrence	PSMA-PET defines all lesions to be treated	PSMA-guided SABR vs. standard care	Ongoing	PSMA-PET determines completeness of focal treatment
PROBE, [39]	Ongoing	RT focal boost	Primary localized PCa	PSMA-PET for boost volume definition	PSMA-PET-guided boost + standard RT	Feasibility and toxicity endpoints	Formal testing of PSMA-PET as boost-defining tool
PRIMARY Trial, [40]	2021	Diagnosis → focal planning	Suspected localized PCa	PSMA-PET + MRI improves lesion detection	Diagnostic accuracy study	PSMA-PET improves detection of clinically significant lesions	Supports PSMA-PET–guided focal therapies
UCLA PSMA-guided focal therapy cohorts, [41]	2020–2022	HIFU/focal laser	Localized PCa	PSMA-PET used to select and target lesions	PSMA-PETguided focal therapy	Improved lesion targeting vs. MRI alone	Supports PSMA-PET for precision focal therapy

HIFU = High-Intensity Focused Ultrasound; RT = radiotherapy; MDT = metastatic directed therapy.

## Data Availability

The original contributions presented in this study are included in the article. Further inquiries can be directed to the corresponding author.
